# Alpha-Smooth Muscle Actin-Positive Perivascular Cells in Diabetic Retina and Choroid

**DOI:** 10.3390/ijms21062158

**Published:** 2020-03-20

**Authors:** Soo Jin Kim, Sang A. Kim, Yeong A. Choi, Do Young Park, Junyeop Lee

**Affiliations:** 1 Department of Ophthalmology, Yeungnam University College of Medicine, Daegu 42415, Korea; freicas@naver.com (S.J.K.); zzanga5897@naver.com (S.A.K.); kei97437@gmail.com (Y.A.C.); doyoung83.park@gmail.com (D.Y.P.); 2 Department of Ophthalmology, Asan Medical Center, University of Ulsan, College of Medicine, Seoul 05505, Korea

**Keywords:** alpha-smooth muscle actin (αSMA), perivascular cell, pericyte, vascular smooth muscle cell, diabetic retinopathy, diabetic choroidopathy

## Abstract

Structural alterations of pericytes in microvessels are important features of diabetic retinopathy. Although capillary pericytes had been known not to have α-smooth muscle actin (αSMA), a recent study revealed that a specific fixation method enabled the visualization of αSMA along retinal capillaries. In this study, we applied snap-fixation in wild type and streptozotocin-induced diabetic mice to evaluate the differences in vascular smooth muscle cells of the retina and the choroid. Mice eyeballs were fixed in ice-cold methanol to prevent the depolymerization of filamentous actin. Snap-fixated retina showed αSMA expression in higher-order branches along the capillaries as well as the arterioles and venules, which were not detected by paraformaldehyde fixation. In contrast, most choriocapillaris, except those close to the arterioles, were not covered with αSMA-positive perivascular mural cells. Large choroidal vessels were covered with more αSMA-positive cells in the snap-fixated eyes. Diabetes induced less coverage of αSMA-positive perivascular mural cells overall, but they reached higher-order branches of the retinal capillaries, which was prominent in the aged mice. More αSMA-positive pericytes were observed in the choroid of diabetic mice, but the αSMA-positive expression reduced with aging. This study suggests the potential role of smooth muscle cells in the pathogenesis of age-related diabetic retinopathy and choroidopathy.

## 1. Introduction

Pericytes along the blood vessels are the specific mural cells embedded within the basement membrane of the vasculature and adjacent to endothelial cells. Perivascular mural cells, including pericytes and vascular smooth muscle cells (VSMC), maintain the structural and functional stability of blood vessels [1]. Compared with large-diameter vessels (e.g., arteries and veins), capillaries have the distinct characteristic that they are not covered by vascular smooth muscle cells [1]. As research advanced, the pericytes were re-established to support not only capillaries but also medium-sized vessels such as arterioles and venules, and often large vessels [2,3]. Pericytes have been found to express alpha-smooth muscle actin (αSMA), a contractile filamentous component which is a major indicator of vascular smooth muscle cells [4,5]. Interestingly, retinal capillaries are affected by vasoregulators, and thus the vascular tone of these capillaries is controlled; however, retinal capillaries lack vascular smooth muscle cells to generate the contractile function [6,7]. Meanwhile, recent studies detected the existence of capillary α-SMA by preventing filamentous actin depolymerization in the retina via the intravitreal injection of phalloidin and ice-cold methanol fixation (Snap fix) [8].

The retina is the bowl-like shaped innermost neural layer of the eye, which gradually thins out toward the periphery. Blood circulates throughout the retinal vasculature, from the central retinal artery to the capillary, and is finally drained out to the central vein; thus, the central area has rather thicker vessels, including arteries and veins, compared with vessels at the peripheral retina [9]. This indicates that the gradient of blood pressure and blood flow is regulated differently in different regions. Diabetic retinopathy (DR) is a diabetes complication that affects eyes which can eventually cause blindness. It is well known that DR causes peripheral non-perfusion and late peripheral leakage, which later progresses to the central macular area, and thereby suggests an abnormal regulation of blood flow in the diabetic retina [10,11]. Nonetheless, topographic differences of perivascular mural cells in the normal and diseased retina have not yet been evaluated. Meanwhile, pericyte loss as pathogenesis of DR has been extensively studied, including our group [12,13,14,15,16,17], and the arteriolar VSMC alteration of DR has been established [18]. However, the αSMA positive mural cell changes in the diabetic retinal capillaries have also not been well elucidated yet.

As the choroidal blood vessels play a critical role in retinal homeostasis, we assumed that choroids in diabetic eyes may show pathological features related to alterations in perivascular cells, which may be associated with diabetic choroidopathy [19,20]. We decided to adapt the Snap fix method not only to the retina but also to the choroid and apply it to detect retinal and choroidal perivascular cells in diabetic models. In this research, we evaluated the validity of using methanol fixation (Snap fix) for the detection of αSMA positive mural cells in both the retina and choroid. Further, we evaluated the change of αSMA positive perivascular cells in diabetic mice models.

## 2. Results

### 2.1. Snap Fixation for the Detection of αSMA Expression in Wild Type Mice

#### 2.1.1. αSMA Positive Perivascular Cells in Large-Diameter Vessels in the Retina and Choroid

**Figure 1:**
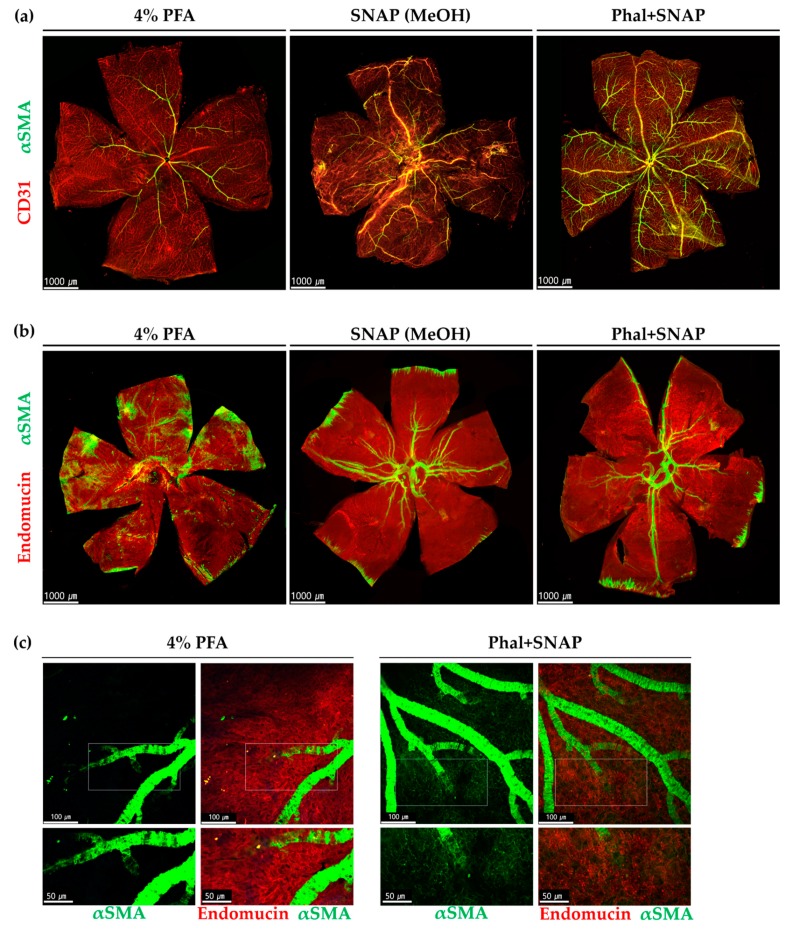
αSMA expression in retinas (**a**) and choroids (**b**,**c**). PFA fixation, snap fixation, and snap fixation after intravitreal injection of phalloidin (Phal+SNAP) were compared. Note that snap fixation or adding phalloidin injection before snap fixation enabled more visualization αSMA positive perivascular mural cells than PFA fixation. (**b**) Choroid showed significantly higher αSMA detection in large vessels when using snap fixation regardless of phalloidin injection. Whereas 4% PFA fixation enabled only a few arterial α, SMA to be detected. (**c**) αSMA expression in the choriocapillaris. More αSMA positive cells were observed at choriocapillaris, mostly post-arteriolar capillaries in Phal+SNAP. The lower picture depicts the magnified area of the indicated square.

To enhance the detection of αSMA, we performed snap fixation with and without intravitreal injection of phalloidin in six-week-old mice. The snap-fixed retina showed significantly increased αSMA expression levels at all vascular networks, including veins, compared with those observed using conventional paraformaldehyde (PFA) fixation (Figure 1a). Phalloidin injection before snap fixation enabled the detection of more αSMA positive perivascular cells in medium- and large-sized vessels of the retina (Figure 1a). To visualize the choroidal vasculature, we transparentized choroids by bleaching the retinal pigment epithelium (RPE) and melanocytes (Figure S1). Snap fixation, regardless of phalloidin injection, showed significant improvement of αSMA detection in large-diameter vessels (Figure 1b).

#### 2.1.2. αSMA-Positive Perivascular Cells in Capillary Networks of the Retina and Choroid

Following the previous studies [8], to analyze the efficiency of snap fixation in capillary networks, we evaluated the average count per order of the αSMA-positive perivascular mural cell expressing branch out from the central retinal artery. We also evaluated the snap fixation at the three capillary plexuses of the retina; superficial capillary plexus (SCP), intermediate capillary plexus (ICP), and deep capillary plexus (DCP). Compared with PFA fixation, snap fixation enabled detection of more branches and higher order, and phalloidin injection further enhanced higher-order detection. Capillary level αSMA-positive perivascular cells were scarce and only detected at the SCP when fixed with PFA. By using snap fixation, however, capillary level αSMA positive perivascular cells were also detected at the ICP and DCP, as well as the SCP. Phalloidin injection further improved the detection with snap fixation, especially at the deep vasculature plexus (Figure S2).

In addition to the retina, we also evaluated the distribution of αSMA expressing perivascular cells in the choriocapillaris. While αSMA-positive cells were rarely detected in the PFA-fixated choriocapillaris, more were observed in the snap-fixated choriocapillaris, mostly in the post-arteriolar capillaries, especially when snap fixed after phalloidin injection. The αSMA expressing perivascular mural cells in the choriocapillaris were located in the outer capillary networks (Figure 1c).

### 2.2. Retinal and Choroidal αSMA in STZ-Induced Diabetic Mouse Model

**Figure 2:**
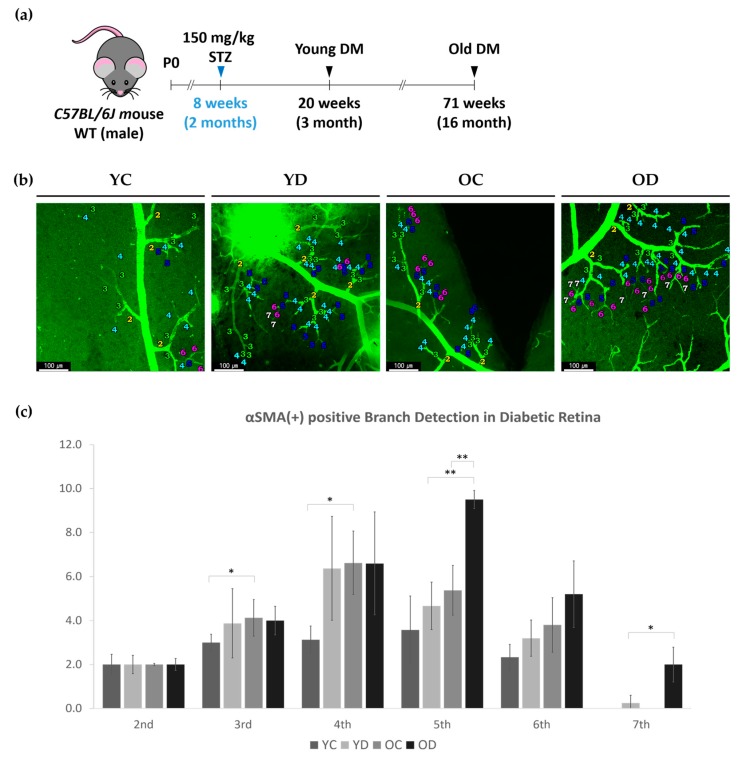
αSMA-positive branch order detection in diabetic retina. (**a**) Diagram depicting the experiment schedule for induction of streptozotocin-induced diabetes at 8 weeks old C57Bl/6 male mice. (**b**) Representative images show the branches including the highest-ordered branch detected in each condition; Young Control (YC), Young Diabetes (YD), Old Control (OC), and Old Diabetes (OD). Note that both diabetic groups were able to detect up to 7th order. (**c**) The graph indicates the counts of average branch orders detected per two second ordered branches. Error bars indicate SD. (2-way ANOVA; *N* = 16 per group, * *p* < 0.05, ** *p* < 0.01).

The older diabetic mice were vulnerable to anesthetics, and hence, we did not perform intravitreal injection of phalloidin before snap fixation to all four groups: young control (YC), young diabetes (YD), old control (OC), and old diabetes (OD). The young groups were 20 weeks old (3 months diabetic duration), and the old groups were 71 weeks old (diabetic duration of 14 months; Figure 2a). We compared three different parameters between specifically selected groups: early phase diabetes in YC and YD, aging effect in YC and OC, and aging and longer diabetic effect in YD and OD, OC and OD. Direct evaluation between YC and OD was not performed.

#### 2.2.1. Regional Changes of Retinal αSMA in the Diabetic Model

We evaluated the branch order of the retinal superficial branches in the YC, YD, OC and OD groups, as previously described. Compared to YC, OC had an increased number of αSMA-positive 3rd and 4th order branches (Figure 2b,c). OD had a higher number of αSMA-positive 5th order branches than YD or OC. We were able to detect αSMA positive branches up to the 7th order branch in both diabetic groups, especially in OD, detecting as much as those detected in the 2nd order branch on average. This result indicates that the αSMA expression along the arteries is affected by aging, but arterioles and capillaries are affected by both aging and diabetes (Figure 2b,c).

**Figure 3:**
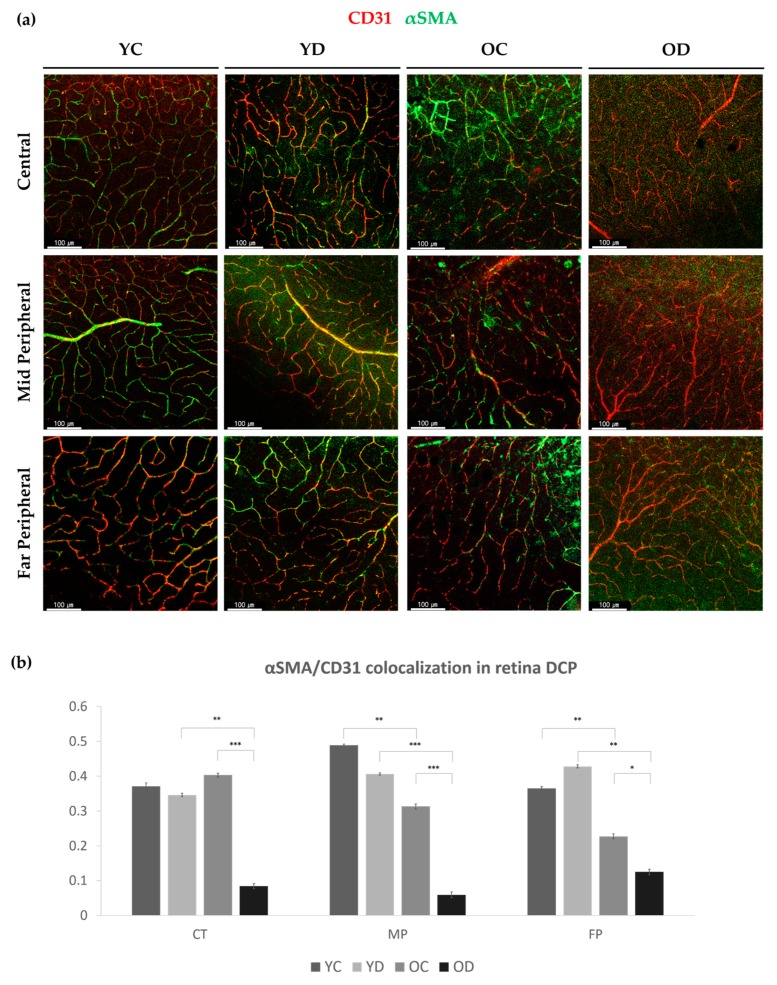
Deep capillary αSMA positive perivascular cells in the diabetic retina. (**a**) Regional distribution difference in retinal deep vessel αSMA ensheathment at the central area, mid-peripheral area and far-peripheral area in YC (Young Control), YD (Young Diabetes), OC (Old Control), and OD (Old Diabetes). (**b**) For each of the groups and regions, αSMA colocalized over CD31 was measured with the Manders coefficient. Note that OD showed significantly reduced αSMA coverage at every region compared to either YD or OC. 4 different samples were analyzed and averaged per condition. Error bars indicate standard deviations (1-way Anova; *N* = 16 per group, * *p* < 0.05, ** *p* < 0.01, *** *p* < 0.001)**.**

However, the αSMA-positive perivascular cells in the deep capillary plexus (DCP) varied among different regions (Figure 3a). Compared with YC, YD showed a trend in which the αSMA-positive perivascular coverage was reduced in the mid-peripheral area but increased at the far-peripheral area with marginal significance. Compared with YC, OC showed a reduced αSMA coverage in the mid- and far-peripheral area. Notably, the OD group presented a global loss of αSMA coverage in all the regions of the DCP (Figure 3b). These results indicate that early diabetes increased αSMA coverage mainly at the far-peripheral retina, whereas with aging, the αSMA expression levels at the mid- and far-peripherals were significantly reduced. This was more evident in the aging group with longer diabetic duration (Figure 3b).

**Figure 4:**
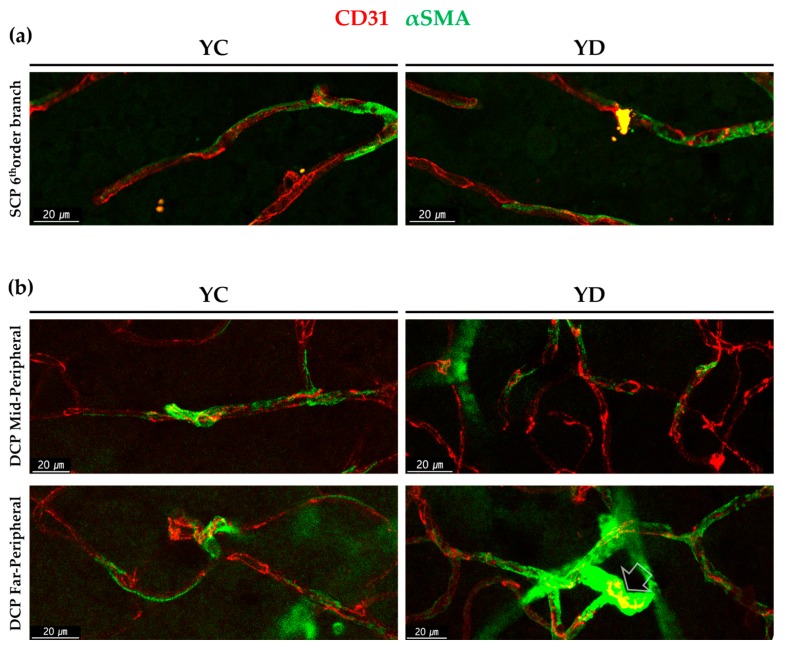
Morphological difference among αSMA positive perivascular mural cells in young diabetic retinal capillaries. (**a**) αSMA positive perivascular mural cells in the 6th order branch in the retinal superficial vessel in YC and YD. (**b**) αSMA positive perivascular mural cells in mid-peripheral and far-peripheral in deep vessels in YC and YD. Note that while YC show overall long, stretching shape, far peripheral YD shows highly dense, fully wrapped around shape at some points (arrow).

The morphological difference among αSMA positive perivascular mural cells in YC and YD were also analyzed. At the 6th order branch in retinal SCP, both YC and YD showed pericyte-like existence as previously studied [8] (Figure 4a). In the case of the DCP, the mid-peripheral regions showed similar morphology between YC and YD similar to the SCP. However, in the far-peripheral DCP, some points of YD showed different morphologies; highly dense, fully wrapped around like VSMC (Figure 4b).

#### 2.2.2. Choroidal αSMA in the Diabetic Model

**Figure 5:**
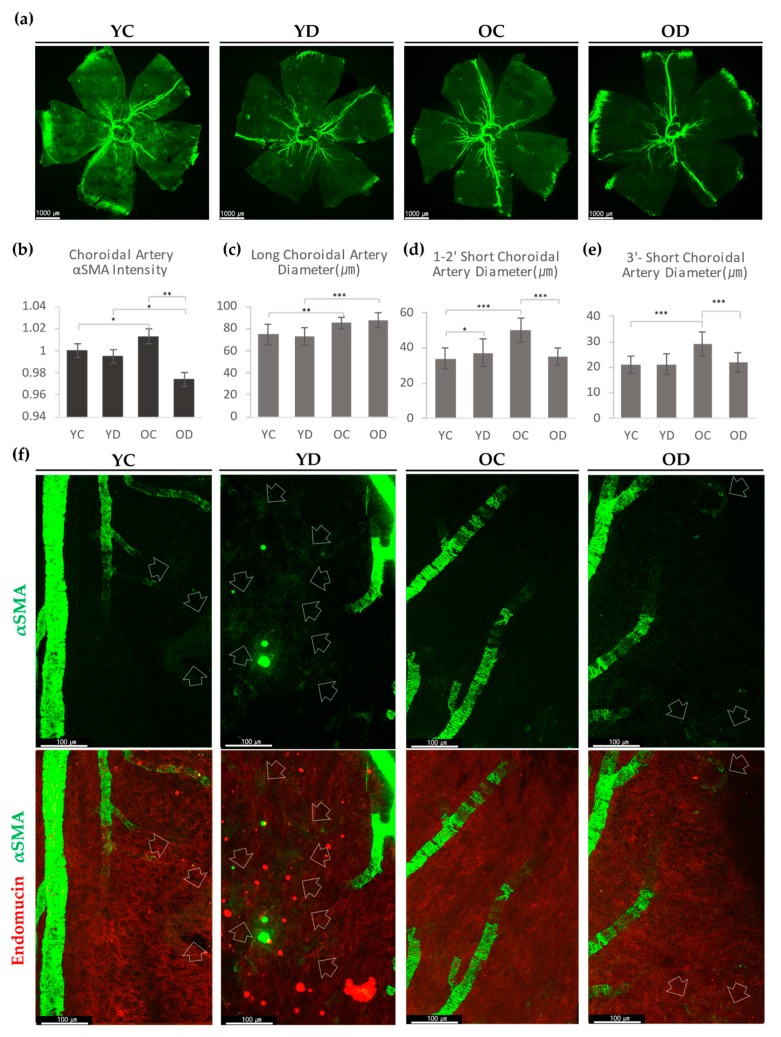
αSMA expression of the diabetic choroid. (**a**) Representative choroidal wholemount images from Young Control (YC), Young Diabetes (YD), Old Control (OC), and Old Diabetes (OD). (**b**) Quantification of relative fluorescence intensity of choroid arterial αSMA. (**c**) The diameter of the long choroidal artery that traverses from central to the far-peripheral edge. Diameters were measured at 8 points per each sample. (**d**) The diameter of 1st and 2nd order short choroidal artery. Diameters were measured at 15 points per each sample. (**e**) The diameter of the 3rd order or higher short choroidal artery. Diameters were measured at 20 points per each sample. (**f**) YD showed remarkably increased αSMA expression at choriocapillaris than YC. OC choroid hardly showed capillary αSMA, where OD revealed αSMA in choriocapillaris but greatly reduced from YD. Arrows indicate the expression of αSMA at choriocapillaris. (**b**–**e**) 4 different samples were analyzed and averaged per condition. Error bars indicate SD. (Student’s *t*-test; *N*_(*b*)_ = 16, *N*_(*c*)_ = 32, *N*_(*d*)_ = 60, *N*_(*e*)_ = 80 per group; * *p* < 0.05, ** *p* < 0.01, *** *p* < 0.001).

As the choroid of the diabetic model has not been well elucidated, we first decided to observe its overall αSMA expression in all four groups and assess any differences at a glance. Because the αSMA is mainly expressed at the smooth muscle cells within large vessels, choroidal arteries were mostly immunostained with αSMA. Both diabetic groups showed a more reduced signal intensity than the control groups (Figure 5a). In the aging results, OC had better αSMA detection in choroidal arteries than YC. However, OD showed a markedly reduced αSMA signal than YD, which was an opposite trend from that of the controls. These results suggest that diabetes downregulates choroidal arterial αSMA expression, even reversing the age-related increment of αSMA (Figure 5b).

αSMA-positive perivascular cells at the choriocapillaris were mainly detected at the post-arteriolar capillaries (near the end of the choroidal arteries). YD noticeably showed more capillary αSMA than YC (Figure 5f). OC, compared with YC, barely expressed capillary level αSMA. In OD, we were able to detect some capillary αSMA cells (Figure 5f). These results demonstrate that diabetes increases choroidal αSMA-positive perivascular mural cells at the post-arteriolar choriocapillaris.

#### 2.2.3. Evaluation of Retinal and Choroidal Vessels in Diabetic Model

**Figure 6:**
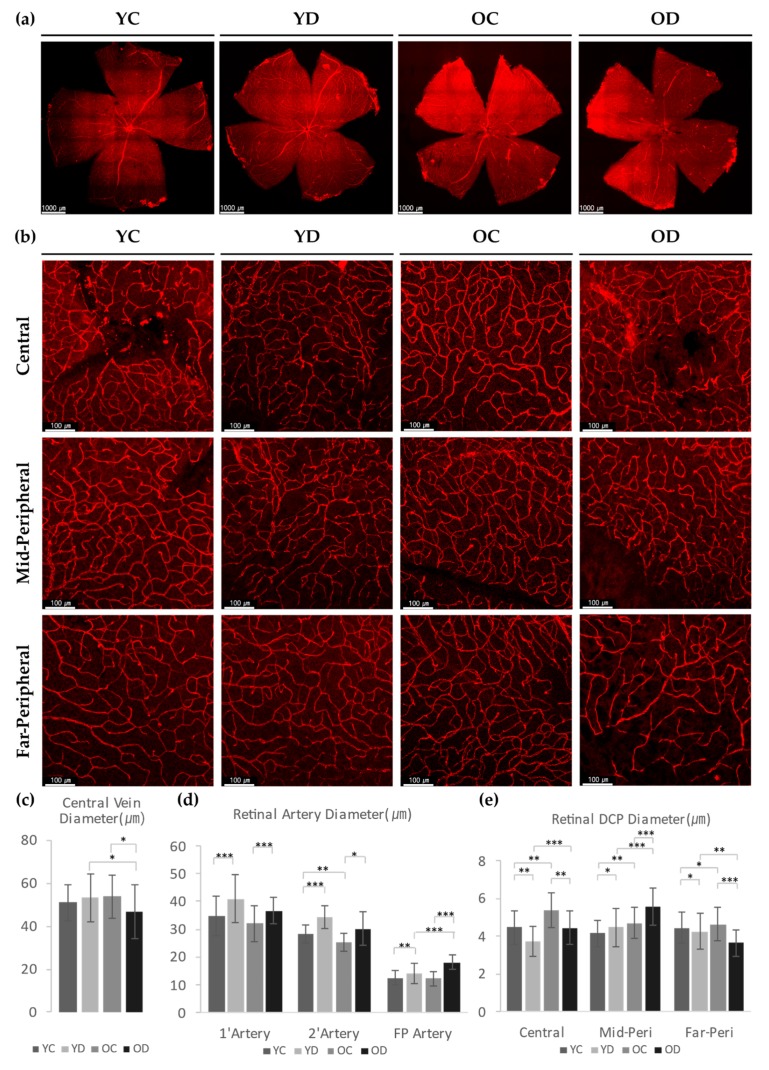
Retinal blood vessels in a diabetic model. (**a**) Wholemount retinas with CD31 staining for blood vessels. (**b**) Retinal deep vessels stained with CD31 at three different regions; central, mid-peripheral, far-peripheral. (**c**) The diameter of the central veins from each group. Each sample was measured with at least 8 different points. (**d**) Retinal artery diameters were analyzed. Each sample was measured with at least 10 different points per arteries of interest. (**e**) Retinal deep capillary diameters from three regions of each group were analyzed. Each sample was measured with at least 20 different points. (**c**–**e**) 4 different samples were analyzed and averaged per group. Error bars indicate SD. ((**c**) Student’s *t*-test, *N*_(*c*)_ = 32 per group; (**d**,**e**) 2-way Anova tested, *N*_(*d*)_ = 40, *N*_(*e*)_ = 80 per group; * *p* < 0.05, ** *p* < 0.01, *** *p* < 0.001).

We first measured the diameter of the retinal arteries and veins of all four groups. The diameter of the central retinal vein in OD was smaller than that of OC or YD (Figure 6a,c). Meanwhile, the retinal arteries in both diabetic groups displayed larger diameters than that of the control groups (Figure 6d). These results imply that diabetes has a greater effect in shaping the size of the retinal arterial diameter than aging.

As the αSMA in the DCP was critically affected by diabetes, we also measured the DCP diameter. At the central region, YD had thinner capillaries than YC, but OD had a larger diameter than YD (Figure 6b,e). The mid-peripheral region of YD revealed dilated and deep capillaries than YC, while that of OD showed marked dilation than that of OC. The far-peripheral region revealed the exact opposite in OD results as OD showed a remarkably reduced DCP diameter. However, YD had a more slightly reduced diameter than YC, while OC had a slightly larger diameter than YC (Figure 6e). These results indicate that aging induced globally dilated DCP in the whole retinal area, while diabetes induces variable DCP diameter changes according to the regional retinal area, which includes thinning of the DCP except in the mid-peripheral area. Collectively, our data suggest that additional factors other than αSMA may regulate the DCP diameter in diabetes, such as vascular inflammation or hemodynamics.

In the choroid, we evaluated the average diameter of the major choroidal arteries: 1st and 2nd order choroidal arterioles, and 3rd and higher-order choroidal arterioles. Both aged groups had thicker major choroidal arteries than the young groups (Figure 5c). In contrast, diabetes did not affect the diameter of major arteries. However, in the choroidal arterioles, OD showed no changes from YD, whereas OC appeared to have a larger diameter than YC (Figure 5d,e). These results demonstrate that long-term diabetes effects in old age can result in significantly reduced αSMA expression in the choroidal arterioles.

## 3. Discussion

In this study, we verified the existence of αSMA-positive perivascular cells in retinal capillaries by performing snap fixation as previously studied [8]. When the samples were fixed with PFA, no αSMA-positive cells were detected in the capillaries, as previously reported [6,21]; however, snap fixation revealed αSMA positive capillary perivascular cells in all three layers, residing along the capillary similar to capillary pericytes [4,22,23] rather than wrapping around like arterial VSMC. Inhibition of F-actin depolymerization was favorable for αSMA detection in the retinal capillary. This may suggest that αSMA-expressing perivascular cells at the capillary level have the characteristic of rapidly depolymerizing actin fiber or quite low levels of contractile actin fiber, thus making them less easily detectable; this is similar to a feature of synthetic VSMC [24,25].

Further, we verified the presence of αSMA expressing perivascular cells in the choroid using snap fixation. Consistent with the previous report demonstrating that the choroidal pericytes located at the outer surface of choriocapillaris [26,27], αSMA-expressing perivascular cells also resided at the scleral side and were especially abundant at the post-arteriolar capillaries. This indicates the possibility of capillary level αSMA-expressing perivascular mural cells as formal choroidal pericytes. However, the morphology of αSMA-expressing perivascular mural cells is more likely flat, similar to synthetic VSMC [28]. In addition, F-actin depolymerization increased αSMA detection in the αSMA-expressing perivascular mural cells, and thus can be considered as due to the low levels of contractile actin fiber in synthetic VSMC, thus were hard to detect with preventing F-actin depolymerization or snap fixation. Despite the similarities, we can suggest, our results can neither classify capillary level αSMA perivascular mural cells as formal capillary pericytes nor as VSMCs, as this needs to be further studied.

However, we shed light on the retinal and choroidal αSMA-expressing perivascular cell function at the capillary level. We detected the capillary level αSMA in diabetic conditions. As diabetic retinopathy is a progressive disease [29], we investigated early and prolonged diabetic-conditioned retinas and choroids. Surprisingly, early phase diabetic retina showed some differences in capillary level αSMA expression compared with old-aged prolonged diabetic retina and littermate controls. In the early phase, short-term diabetic retina showed increased superficial capillary αSMA-positive perivascular mural cell expression in the detection of the 7th order αSMA-positive retinal branches. In the late phase, the long-term diabetic retina exhibited tremendous alterations in αSMA-expressing perivascular mural cells of superficial capillaries, showing significantly higher detection in the 7th order branches. As the 7th order branches can be considered as capillaries, we can view this phenomenon as a transition of the pericyte or alteration of the pericyte’s characteristics, like the VSMC phenotype transition.

In the non-diseased group, the deep retinal capillary diameter was similar to that at a younger age but progressed to be overall dilated when aged. In the short-term diabetic group, retinal deep capillary diameter in the central and far-peripheral retina is less than in the mid-peripheral retina; as age progresses, it increases in the central and mid-peripheral retina but decreases in the far-peripheral retina with prolonged DM. Therefore, the distribution of αSMA positive perivascular cells does not exactly match with the regional diameter of DCP in diabetes. These suggest that factors other than αSMA, such as vascular inflammation or hemodynamics, may regulate DCP diameter in diabetes. These tendencies cannot be determined with αSMA alteration alone but also require pericyte dropout in diabetic retinal capillary, as previously studied [1,2,13,17]. However, the regional difference of pericyte distribution or depletion in the diabetic retina deep vessel has not been elucidated, thus the full consideration is yet to be reported. Nevertheless, we could consider that diabetic condition alters the regional pericyte coverage as observed in αSMA expressing perivascular mural cells in our study.

In the early phase of the short-term diabetic retina, the αSMA expression alteration in the DVP varied along the region as it decreased in the mid-peripheral retina and increased in the far-peripheral regions. The role of αSMA as a contractile actin fiber could be the explanation for the increased diameter of the mid-peripheral deep vessel and the reduced diameter of the far-peripheral deep vessel. In addition, the retinal blood flow in the DCP could be vulnerable to αSMA-mediated capillary contraction and ischemia in the far-peripheral retina [30]. In contrast, in the late phase, long-term diabetic retina showed distinct alterations in the αSMA-expressing perivascular mural cells in deep capillaries. With aging, the αSMA expression levels at the mid-and far-peripherals were significantly reduced, which was more evident as age progressed with longer diabetic duration. This implied that long-term old DR DCP is not benefitted by αSMA ensheathment, and thus blood flow at peripheral retina maintains passive regulation by SCP.

The diabetic choroidal vessels also showed a distinct differential trend compared to the retina in our data. Our study showed increased αSMA-expressing perivascular mural cells in the choriocapillaris in diabetes. We speculate these αSMA-positive cells could be either non-vascular smooth muscle cells or vascular smooth muscle cells which are increased by endothelial-mesenchymal transition through the hyperglycemia-induced inflammation [31]. However, due to the uncertain morphology of the choriocapillaris perivascular mural cells we detected, we came up with the necessity of future studies on choroidal pericytes and choriocapillaris level VSMCs to completely understand this αSMA-positive perivascular mural cell’s characteristics. The expression levels of αSMA in choroidal large-diameter vessels were more complex. In healthy groups, the arterial αSMA expression increased with aging, but this expression decreased with aging in diabetic groups. The ICG angiography of type 2 diabetic patients shows dilated choroidal vessels, which is called diabetic choroidopathy [20]. Our study showed decreased αSMA expression in major choroidal arteries in diabetes, and therefore, we suggest that the loss of αSMA expression in major aged choroidal arteries are involved in the dilation of large choroidal vessels because most patients with diabetic choroidopathy are from an aged population [20].

Meanwhile, we can consider that the arterial αSMA is expressed in arterial VSMCs, and thus interpret that the choroidal artery diameter with the increased thickness of tunica media by increased αSMA expression and decreased tunica media thickness by decreased αSMA expression. Our data suggest that the long-term diabetic choroid artery may contain insufficient arterial VSMC for aging compensation or can be free from an age-dependent stiffed artery. In this respect, we speculate the possibility that diabetic choroidopathy could be associated with the progression of age-related macular degeneration as diabetes prevents choroidal arterial stiffness by downregulating αSMA in large choroidal vessels. To elucidate the physiological significance of choroidal pericytes and non-vascular smooth muscle cells, further studies on the loss of function using animal models and clinical correlations are required.

In summary, we focused on the detection of αSMA-expressing perivascular mural cells in both the retina and the choroid using the snap fixation method. Diabetes induced less coverage of αSMA-positive perivascular mural cells but reach to the higher-order branches of retinal capillaries, which was prominent in the aged mice. In the choroid, more αSMA-positive perivascular mural cells were observed in the diabetic mice; however, the expression levels decreased with aging. This study suggests the potential role of vascular smooth muscle cells in the pathogenesis of age-related diabetic retinopathy and choroidopathy.

## 4. Materials and Methods

### 4.1. Normal Mice and STZ-Induced Diabetic Mice Model

Animal care and experimental procedures were approved by the Animal Care Committee of Yeungnam University College of Medicine (Project ID: YUMC-AEC2019-017; Date of approval: 24 June 2019). Mice were handled in accordance with the NIH guide for the care and use of laboratory animals. Specific pathogen-free C57BL/6J mice were purchased from The Jackson Laboratory (Bar Harbor, ME, USA). For the evaluation of the fixation methods for αSMA detection, we used six-week-old C57BL/6J mice.

The diabetic model was generated with STZ injection. At eight weeks old, the mice were injected intraperitoneally (IP) with a single injection of streptozotocin (STZ S0130, Invitrogen, Waltham, MA, USA) at 150 mg/kg to induce diabetes. A blood glucose concentration that exceeded 500 mg/dL was considered diabetic. The control group was injected with a sodium citrate buffer. We divided the experimental mice into four groups: young control (YC), young diabetes (YD), old control (OC), and old diabetes (OD). The young groups were 20 weeks old (three months DM duration), while the old groups were 71 weeks old (DM duration of 14 months).

### 4.2. Intravitreal Injection of Phalloidin

To prevent actin depolymerization, F-actin was fixed in vivo by 3 µL intravitreal injection of phalloidin (00028, Biotium, Fremont, CA, USA) using a custom ordered microneedle (Inner diameter 60 µm, Outer diameter 120 µm, length 3 mm, Incyto, Cheonan, ROK). Mice were anesthetized before injection with an intraperitoneal injection of 6 mg/kg ketamine and 6 mg/kg xylazine. After 2.5 h, mice were sacrificed and the eyeballs were collected and fixed in −20 °C methanol for 1 h. Retinas were harvested and subjected to the immunofluorescence staining protocol.

### 4.3. Wholemount Immunofluorescence and Confocal Microscopy of the Retina

The eyeballs of sacrificed mice were collected and fixed immediately with ice-cold methanol for 1 h at −20 °C or with 4% PFA overnight. The fixed eyeballs were carefully trimmed to remove the lens and RPE-choroid. Immunofluorescence staining of whole-mounted retinas and choroids were performed as previously described, with few modifications [32,33]. The retinas were incubated in a blocking solution for 1 h at room temperature and then incubated with the respective primary antibodies (CD31; BD 550274; Franklin Lakes, NJ, USA, Endomucin; Santacruz SC65495; Dallas, TX, USA, Anti-Actin ACTA2; Sigma-Aldrich A5228; St. Louis, MO, USA) diluted in blocking solution (1:200) at 4 °C overnight. Samples were washed in 0.2% PBST 3 times for 5 min and incubated with secondary antibody diluted in 0.1% PBST (1:1000) at 4 °C overnight. Afterward, the samples were washed 3 times for 5 min, mounted with Prolong Glass Antifade Mountant (Invitrogen P36980; Waltham, MA, USA), and were imaged and analyzed with a confocal laser scanning microscope (Leica TCS SP2 and TCS SP8X; Wetzlar; Germany). Three-dimensional vascular images and projected images were produced from a set of Z-series images by using the accompanying software LasX(v.3.6.0).

### 4.4. Image Analysis

The morphometric and colocalization analyses performed on the retina and the choroid were conducted using Java-based imaging software (ImageJ, v.1.52p, in the public domain at http://rsb.info.nih.gov/ij; National Institutes of Health (NIH), Bethesda, MD, USA, and FIJI [34], an open-source software ImageJ focused on the biological-image analysis). We analyzed the efficiency of snap fixation in capillary plexuses by evaluating the average count per order of the branch out of the retinal central artery. We counted the branches of each order and averaged them per two 2nd order branches to compare αSMA detection in higher-order branches. For the evaluation of the retinal vessel diameter, the retinal artery was measured from the center next to the optic nerve before the main fork (i.e., the division point of the central primary artery branch). Far-peripheral arterioles were measured at each region, and the central vein were measured from the center to the edge. For colocalization analysis, we used the JaCoP plugin for FIJI. To reduce background sensitivity, threshold value were set with the highest auto-threshold-valued YC sample as the default value and applied to all other samples. We picked Manders Coefficient for target-background selective colocalization evaluation; coefficient M_2_ for green channel above red channel [35].

### 4.5. Statistical Analysis

Mice samples were not randomized during the experiments and were not excluded from the analyses. The investigators were not blinded to group allocations during the experiments and outcome analyses. The values are presented as mean ± SD (standard deviations). The statistical significance was calculated using the Student’s *t*-test and 1-way and 2-way ANOVA and Tukey’s multiple comparison tests with PASW Statistics 18 (SPSS v.23). Statistical significance was set to *p* < 0.05.

## Supplementary Materials

Supplementary materials can be found at https://www.mdpi.com/1422-0067/21/6/2158/s1. Figure S1: Induction of transparent choroid by bleaching of choroidal melanocytes and RPE pigments in C57Bl/6 mouse. Figure S2: αSMA expression in capillaries of the retina and choroid.

## Abbreviations

αSMA	Alpha-Smooth Muscle Actin
DCP	Deep Capillary Plexus
DM	Diabetes Mellitus
DR	Diabetic Retinopathy
DVP	Deep Vasculature Plexus, includes ICP and DCP
F-actin	Filamentous Actin, Actin Filament
ICP	Intermediate Capillary Plexus
OC	Old Control
OD	Old Diabetes
PFA	Paraformaldehyde
Phal	Phalloidin
RPE	Retinal Pigment Epithelium
SCP	Superficial Capillary Plexus
VSMC	Vascular Smooth Muscle Cell
YC	Young Control
YD	Young Diabetes
