# Supplementary material for: Alpha-Smooth Muscle Actin-Positive Perivascular Cells in Diabetic Retina and Choroid
Source: Int J Mol Sci. 2020 Mar 20;21(6):2158. doi: 10.3390/ijms21062158 (PMC7139401; doi:10.3390/ijms21062158)
Supplement: Supplementary file 1 [file ijms-21-02158-s001.pdf]

International Journal of *Molecular Sciences*

Supplementary Materials

Alpha-Smooth Muscle Actin-Positive Perivascular Cells in Diabetic Retina and Choroid

Soo Jin Kim [1], Sang A. Kim [1], Yeong A. Choi [1], Do Young Park [1], and Junyeop Lee [2,*]

[1] Department of Ophthalmology, Yeungnam University College of Medicine, Daegu, Republic of Korea; freicas@naver.com (S.J.K.); zzanga5897@naver.com (S.A.K.); kei97437@gmail.com (Y.A.C.); doyoung83.park@gmail.com (D.Y.P.)

[2] Department of Ophthalmology, Asan Medical Center, University of Ulsan, College of Medicine, Seoul, Republic of Korea

* Correspondence: jleeamc@gmail.com (J.L.); Tel.: +82-2-3010-3680

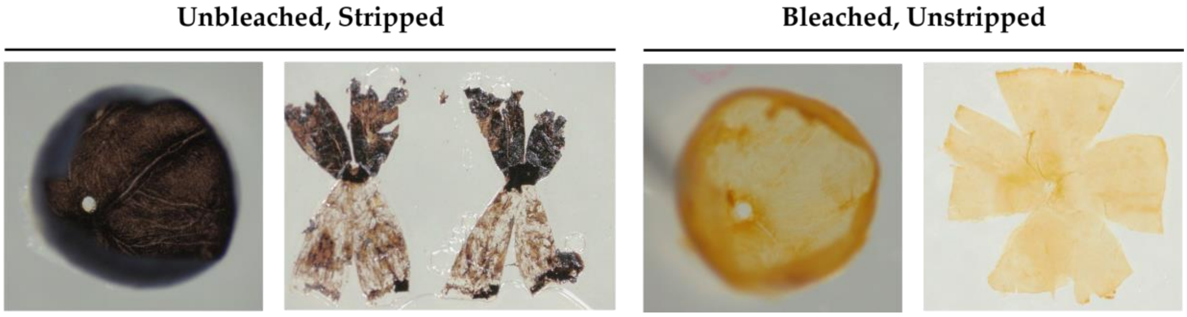

Figure S1. Induction of transparent choroid by bleaching of choroidal melanocytes and RPE pigments in C57Bl/6 mouse. Left shows a previously-used method; RPE-choroidal layer was stripped from sclera. Right shows a bleaching method; choroid exposed to 3% hydrogen peroxide at 4 °C for 5 days. The bleaching method does not require any stripping but have some limitations that they may lose affinity or activity of certain types of surface markers and autofluorescence.

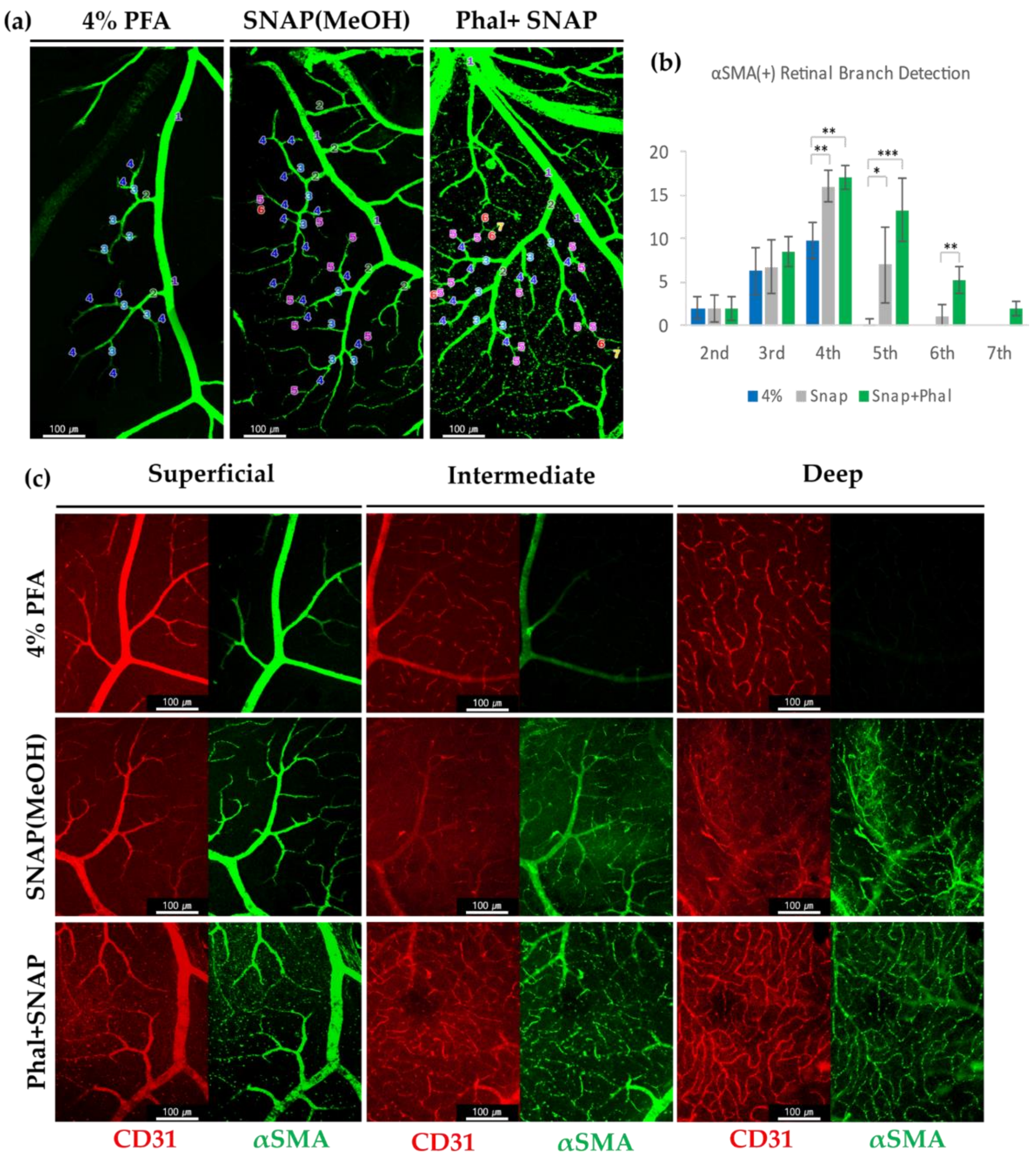

Figure S2. αSMA expression in capillaries of the retina and choroid. (a) αSMA-positive branch order in the retina were compared among PFA fixation, snap fixation, and snap fixation after intravitreal injection of phalloidin (Phal+SNAP) (b) Quantifications of αSMA-positive retinal branch detection. To properly compare the order of the detected branch, the number of branches were computed as counts per two 2nd-order branches. Error bars indicate SD. (2-way Anova; N=10 per group, *$p < 0.05$, **$p < 0.01$, ***$p < 0.001$) (c) αSMA detection in the three different capillary plexuses of the retina.
